# Linear and nonlinear thermoelectric transport in a quantum spin Hall insulators coupled with a nanomagnet

**DOI:** 10.1038/s41598-022-16043-3

**Published:** 2022-07-14

**Authors:** Rui Wang, Hui Liao, Chun-Yan Song, Guang-Hui Tang, Ning-Xuan Yang

**Affiliations:** Department of Physics, College of Sciences, Shihezi University, Shihezi, 832000 China

**Keywords:** Physics, Condensed-matter physics

## Abstract

Thermoelectric effects in quantum systems have been focused in recent years. Thermoelectric energy conversion study of systems with edge states, such as quantum Hall insulators and quantum spin Hall insulators, is one of the most important frontier topics in material science and condensed-matter physics. Based on the previous paper (Gresta in Phys Rev Lett 123:186801, 2019), we further investigated the linear and nonlinear thermoelectric transport properties of helical edge states of the quantum spin Hall insulators coupled with double nanomagnet, calculated the Seebeck coefficients $S_c$ and the thermoelectrical figure of merit *ZT*, discussed the influence of the length of the nanomagnet and the relative tilt angle of component of the magnetization perpendicular on the thermoelectric coefficients ($S_c$ and *ZT*), and summarized some meaningful conclusions in the linear response regime. In the nonlinear regime, we calculated the equivalent figure of merit $ZT_M$ and the power-generation efficiency $\eta$ in different length of the nanomagnet, obtain the temperature difference of achieving optimal thermoelectricity. The results of this paper further confirm that the setup can indeed be used as a device for achieving high performance thermoelectric.

## Introduction

Heat energy and electric energy are common forms of energy in the nature. Turning the waste heat into usable electric energy can not only alleviate the energy crisis, but also reduce environmental pollution^1,2^. It was early discovered that thermoelectric effect enables the interconversion between heat and electric energy^1–8^. Thermoelectric effects include the Seebeck effect, the Peltier effect, and the Thomson effect. Among these, the Seebeck effect is the thermalgradient-induced bias in a two-probe system, which describes a longitudinal thermoelectric effect^6,7,9–12^. Based on the thermoelectric effect, the thermoelectric energy conversion technology was developed to achieve thermoelectric power generation or thermoelectric refrigeration. Manufactured thermoelectric-devices can be widely used in industrial, space probes, military and medical equipment, and so on^1,13,14^.

To achieve applicable thermoelectric-setup, it requires efficient thermoelectric conversion materials. The conversion efficiency of thermoelectric material depends on its thermoelectric figure of merit *ZT*. *ZT* is defined as $ZT=G{{S_c}^2}{\mathcal {T}}/\kappa$ (*G* is the electric conductance, $S_c$ is the Seebeck coefficient, $\kappa$ is the thermal conductance, and ${\mathcal {T}}$ is the absolute temperature of the device)^12,14–17^. The larger $S_c$ and *G*, the smaller $\kappa$, the higher *ZT* value of the thermoelectric material, the better its performance. However, due to the restriction of the Mott relation and the Wiedemann–Franz law^3,18^, $S_c$, *G* and $\kappa$ are interrelated and cannot be regulated alone. In general, the increase of the charge carriers improved the electric conductance *G* , but it also causes the decrease of the Seebeck coefficient $S_c$ and the increase of the thermal conductance $\kappa$, that is to say, any parameter will produce the corresponding parameter offset effect, so that the increase of *ZT* is not obvious^1–3^. Therefore, it is necessary to get the optimal *ZT* from the global perspective.

Most of the previous work of thermoelectric transport in nanoscale systems focused on the linear response mechanism of electric and thermal current to the electric potential or temperature difference, and the linear response theory and Onsager symmetry relations are used to obtain linear response coefficients such as electric conductance, thermal conductance and thermal power $S_c$. However, in the linear regime, since the temperature difference $\delta {\mathcal {T}}$ is much smaller than the temperature ($\delta {\mathcal {T}}\ll {\mathcal {T}}$), the efficiency $\eta$ remains very low even if thermoelectric figure of merit *ZT* can be very large, $\eta \approx \delta {\mathcal {T}}/{\mathcal {T}}\ll 1$^19–21^. Nonlinear effects in nanoscale systems require the application of large driving forces at small distances, and nonlinearity has been predicted to cause thermal rectification effects^22,23^ and low temperature cooling^24^. A deep understanding of nonlinear effects is needed to evaluate the thermal performance of thermal engines and multi-terminal thermal-electric conversion devices. Therefore, it is important to investigate the nonlinear thermoelectric properties of the nanosystems^19–21^.

Topological insulator is a topological material newly discovered in recent years. It has an insulating body state and a conductive metallic surface state. The surface state is protected by the time-reversal symmetry of nonmagnetic impurity scattering^25,26^. Many studies have shown that topological insulators have excellent thermoelectric properties and can be used as potentially highly-efficient thermoelectric materials. For example, Ma et al.^27^ studied the thermoelectric transport properties of the three-dimensional topological insulator thin film and found that thermoelectric coefficients exhibit rich behaviors. Zhang et al.^28^ found that the thermoelectric effect of the topological insulator thin films is mainly determined by the bulk states. Yang et al.^12^ studied the thermoelectric properties of the surface states in three-dimensional topological insulator nanowires and found that thermoelectric coefficient is strongly dependent on the gate voltage and the magnetic fields. Chen et al.^29^ found that the optimal *ZT* in the topological phase transition region of  the topological insulator. These studies have provided a new idea for performance optimization of thermoelectric materials.

**Figure 1:**
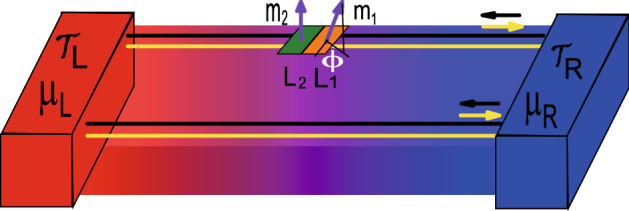
Schematic diagram of a QSHI system connected to a hot and a cold lead. Two nanomagnets with magnetic moments $m_1$ and $m_2$ and lengths $L_1$ and $L_2$ are contacted to a helical Kramers pair of edge states. A thermal gradient $\Delta {\mathcal {T}}={\mathcal {T}}_L-{\mathcal {T}}_R$ is applied. We consider $\Delta {\mathcal {T}}={\mathcal {T}}_L-{\mathcal {T}}_R>0$ and $\Delta \mu =\mu _L-\mu _R$ and set ${\mathcal {T}}={\mathcal {T}}_R$.

**Figure 2:**
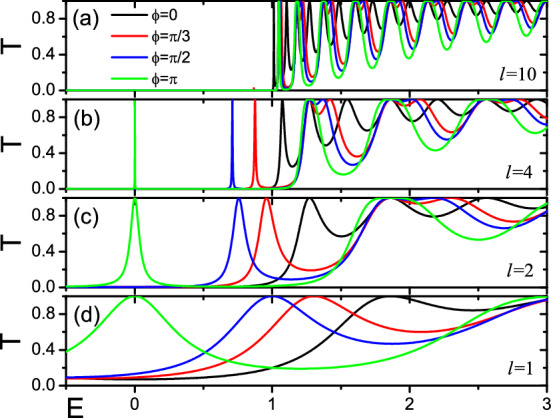
Transmission function for two magnetic domains of equal size (*l*=$L/L_{0}$= 10, 4, 2, 1), with the perpendicular component of the magnetic moments oriented with a relative tilt $\phi$. Energies ($E=\varepsilon / \varepsilon _{\bot }$) are expressed in units of $\varepsilon _{\bot }=Jm_{\bot }$, and lengths are expressed in units of $L_{0}=\varepsilon _{\bot }/\hbar v_{F}$.

The quantum spin-Hall effect (QSH) was first theoretically predicted to exist in Graphene and two-dimensional semiconductor systems^30,31^. QSH state is a completely new matter state which is different from the quantum Hall state, and its implementation does not require an external magnetic field. QSH state has helical edge states, preserves time-reversal invariance. In the QSH system, the bulk state is insulated with an independent energy gap between the conduction and valence band, and the gapless edge states are topologically protected from impurities, which is actually the two-dimensional topological insulators^32–34^. Recently, based on the existence of quantum point contacts and quantum dots, several devices of heat engines and refrigerators have been proposed by using nature of the quantum Hall edge states. Such as: Sánchez et al.^35^ studies the thermoelectric properties of the three-terminal quantum Hall conductor and determines the contribution of the thermoelectric response dependent on the chirality of the carrier motion rather than spatial asymmetry. Roura-Bas et al.^36^ investigated the thermoelectric response of a quantum dot embedded in a constriction of a quantum Hall bar. By applying the gate voltage and the magnetic field on the quantum dot, different thermoelectric working modes can be induced in the device. Takahashi et al.^37^ studied the thermoelectric properties of two-dimensional quantum spin Hall systems and found that edge-state transport is dominant in low-temperature thermoelectric transport. Roura-Bas et al.^38^ studied the thermoelectric response of a setup containing a pair of helical edge states and found that different thermoelectric operational modes can be induced by using the gate voltage and magnetic field. Gresta et al.^39^ studied the thermoelectric properties of a Kramers pair of helical edge states of the quantum spin Hall effect coupled to a nanomagnet and found that this device can achieve high-performance thermoelectric transport in the linear response regime. However, since the temperature difference is less than the temperature, the efficiency $\eta$ remains low in the linear region, even if *ZT* can be very large. In terms of practical usage, devices need to be run at finite power output, where the linearization may not work anymore. A deeper study of the nonlinear thermoelectric transport is needed to accurately assess the thermoelectric properties of the device. Based on the work of Gresta et al.^39^, we studied the linear and nonlinear thermoelectric transport of helical edge states of the quantum spin Hall effect coupled to a nanomagnet by using the device shown in Fig. 1, and analyzed the impact of the length of the two nanomagnets and the relative tilt angle of component of the magnetization perpendicular on the thermoelectric coefficient. In contrast to the work of Gresta et al.^39^, we tended to focus on the nonlinear thermoelectric transport properties in a quantum spin Hall insulator coupled to nanomagnets, and obtained the temperature difference of achieving optimal equivalent figure of merit $ZT_M$ and power-generation efficiency $\eta$. Finally, it has been further proved that this setup can be used as an efficient and useful thermoelectric device, and it is very attractive and promising for the application of the thermoelectricity.

**Figure 3:**
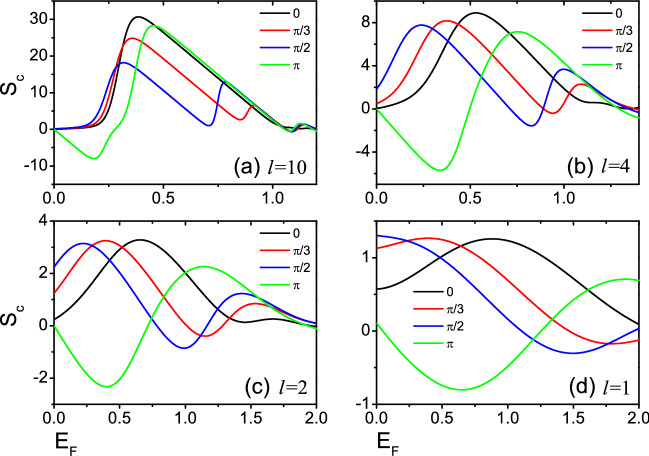
The Seebeck coefficients $S_c$ as functions of Fermi energy $E_F$ for several relative tilt $\phi$ with temperature ${\mathcal {T}}$/${\mathcal {T}}_0$ = 0.02 in (**a**), and ${\mathcal {T}}$/${\mathcal {T}}_0$ = 0.05 in (**b**), and ${\mathcal {T}}$/${\mathcal {T}}_0$ = 0.1 in (**c**) and ${\mathcal {T}}$/${\mathcal {T}}_0$ = 0.05 in (**d**). Two magnetic domains of equal size *l*= 10, 4, 2, 1. The temperatures are expressed in units of ${\mathcal {T}}_0$ =$\varepsilon _{\bot }/k_{B}$.

**Figure 4:**
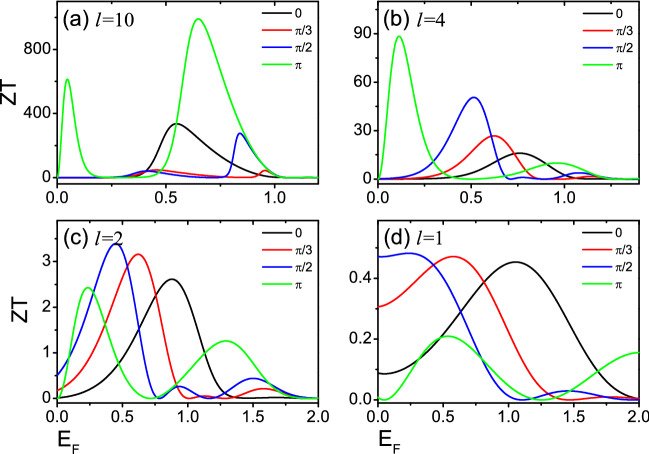
The thermoelectric figure of merit *ZT* as functions of Fermi energy $E_F$ for several relative tilt $\phi$ with temperature ${\mathcal {T}}$/${\mathcal {T}}_0$ = 0.02 in (**a**), and ${\mathcal {T}}$/${\mathcal {T}}_0$ = 0.05 in (**b**), and ${\mathcal {T}}$/${\mathcal {T}}_0$ = 0.1 in (**c**) and ${\mathcal {T}}$/${\mathcal {T}}_0$ = 0.05 in (**d**). The other unmentioned parameters are the same as in Fig. 3.

## Results

### Thermoelectric properties in linear response

Firstly, the transmission function *T*(*E*) was calculated with Eq. (4), and the change of *T*(*E*) is showed in Fig. 2. In the calculation, the length of the two nanomagnets of equal size is taken as *l*=$L/L_{0}$= 10, 4, 2, 1 respectively. The relative tilt angle of magnetic moment perpendicular component orientation of one of the nanomagnet is $\phi _{1}=0$, the other is $\phi _{2}=\phi$. As can be seen from Fig. 2, when the $l=10$, a gap opens in the spectrum of the helical edges. The transmission function tends to a step function, and there is no resonance in the opening of the gap. When the length of the magnetic domains is taken $l=4$, 2 and 1, there is a resonance in the magnetic domains. The resonance position of different relative tilt $\phi$ is different. The width of the resonance decreases with *l* increases. These results are in full agreement with Ref.^39^, but it is different that the current results of $l=4$ and $l=2$ calculations are fully consistent with the trends in $l=10$ and $l=4$ in Ref.^39^ respectively.

Next, we focus on the linear thermoelectric properties of the devices of Fig. 1. Figures 3 and 4 show the Seebeck coefficient $S_c$ and the thermoelectric figure of merit *ZT* versus the Fermi energy $E_F$ for several relative tilt $\phi$. Two magnetic domains of equal size *l* are taken 10, 4, 2 and 1 respectively. $S_c$ and *ZT* exhibit a series of peaks at low temperatures. When $l=4$, 2, 1, the existence of resonance needed to be noticed in Fig. 2, while the resonance width increases gradually with the decrease of *l*, and the corresponding thermoelectric coefficients ($S_c$ and *ZT*) decrease gradually in Figs. 3 and 4. For $l=10$, no resonance is present in Fig. 2, but both $S_c$ and *ZT* is very large in Figs. 3 and 4. The value of *ZT* at the highest peak exceeds 800 at low temperature. This is determined by the open gap in the spectrum of the helical edges. To obtain a larger *ZT* value, it is necessary to have high conductivity *G* to reduce electron heating, high Seebeck coefficient $S_c$ to ensure the output voltage and low thermal conductivity $\kappa$ to maintain large temperature difference. Generally speaking, these three parameters are related to the energy band structure of the material. When the system has an energy gap and the Fermi energy is near the conduction band edge, it will lead to a small thermal conductivity and a large Seebeck coefficient. As a result, *ZT* has a large value^12,40–42^. This suggests that the opening of a gap in the spectrum of the helical edges is very beneficial to the thermoelectric transport of the device, and similar conclusions are shown in Ref.^39^. Furthermore, it is seen from Fig. 4 that the *ZT* value of $\phi =\pi$ for *l*=10, 4 is much greater than other values of $\phi$. This is because a large energy difference between the peaks is observed for the two nanomagnets with $\phi =\pi$, and the first peak after the closing of the gap leads to higher *ZT* peaks. For *l* = 2, 1, the *ZT* values are small, so the height of *ZT* peaks varies slightly for several relative tilt $\phi$.

**Figure 5:**
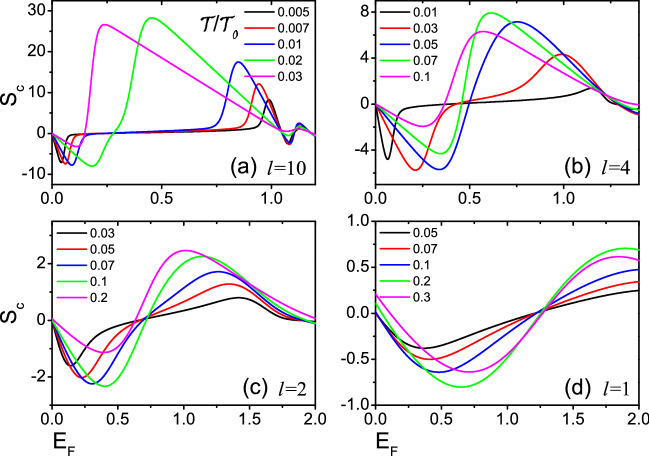
The Seebeck coefficients $S_c$ as functions of Fermi energy $E_F$ for different temperatures ${\mathcal {T}}$/${\mathcal {T}}_0$ with relative tilt $\phi =\pi$. The temperatures are expressed in units of ${\mathcal {T}}_0$ =$\varepsilon _{\bot }/k_{B}$. Two magnetic domains of equal size *l* = 10 in (**a**), and *l* = 4 in (**b**), and *l* = 2 in (**c**) and *l* = 1 in (**d**).

**Figure 6:**
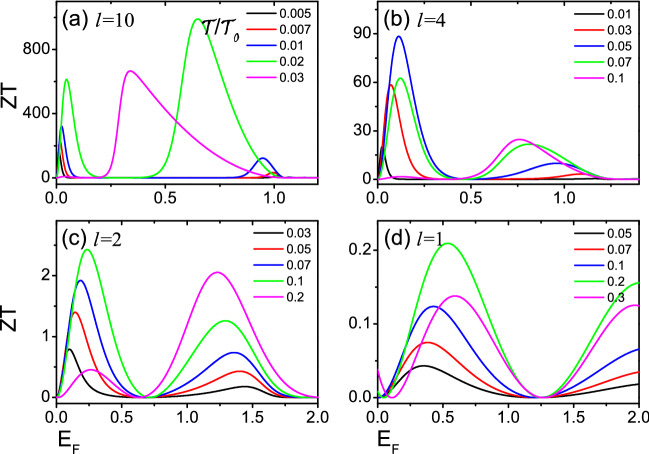
The thermoelectric figure of merit *ZT* as functions of Fermi energy $E_F$ for different temperatures ${\mathcal {T}}$/${\mathcal {T}}_0$ with relative tilt $\phi =\pi$. Two magnetic domains of equal size *l* = 10 in (**a**), and *l* = 4 in (**b**), and *l* = 2 in (**c**) and *l* = 1 in (**d**). The other unmentioned parameters are the same as in Fig. 5.

At last, we study how the temperature affects the thermoelectric transport in detail. When $\phi =\pi$, the values of $S_c$, and *ZT* are larger than those of other relative tilt $\phi$ (see Figs. 3, 4). So, Figs. 5 and 6 show the Seebeck coefficient $S_c$ and the thermoelectric figure of merit *ZT* versus Fermi energy $E_F$ at different temperatures with relative tilt $\phi =\pi$. For *l*=10, $S_c$ display a large peak at low temperatures with $0<E_F<1$, with the corresponding *ZT* also has a large value (see Figs. 5a, 6a). This is because there is no resonance with $0<E_F<1$, and the open energy gap implies that the transmission function $T(E)=0$. A large bias which is required to balance the thermal forces acting on the charge carriers has resulted in very large $S_c$, and *ZT*. For $l=4$, 2 and 1, there is a resonance in the magnetic domains, and the width of the resonance decreases for increasing *l*. The first peak of resonance after the closing of the gap would generate a large thermoelectric coefficients ($S_c$, and *ZT*)^39^. For $E_{F}\sim k_{B}{\mathcal {T}}$, the resonance within the gap affects the thermoelectric response coefficients, and leads to larger *ZT*. *ZT* values is strongly associated with the width of the resonance^39^. For the higher temperatures, when the transport behavior is controlled by the Heaviside function^39^, the thermoelectric coefficient *ZT* is affected by several peaks and the value of *ZT* decreases.

### Thermoelectric properties in nonlinear regime

**Figure 7:**
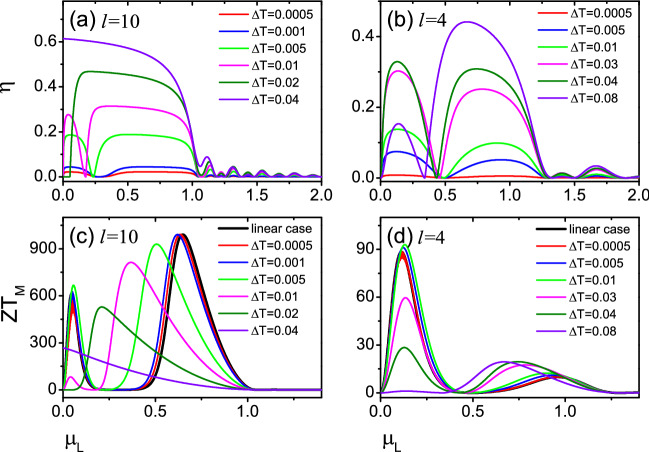
The efficiency $\eta$ and the equivalent figure of merit $ZT_M$ as a function of the chemical potential $\mu _L$ for different temperature gradient $\Delta {\mathcal {T}}$ , with the background temperature ${\mathcal {T}}_R={\mathcal {T}}$ = 0.02 in (**a,c**); ${\mathcal {T}}_R={\mathcal {T}}=0.05$ in (**b,d**). Two magnetic domains of equal size *l* = 10 with $\phi =\pi$ in (**a,c**); *l* = 4 with $\phi =\pi$ in (**b,d**). The thick black lines in (**c,d**) are *ZT* in the linear regime. The other unmentioned parameters are the same as in Fig. 3.

In this section, we will focus on studying the thermoelectric transport properties of this device in the nonlinear regime, computing the equivalent figure of merit $ZT_M$ and the power-generation efficiency $\eta$. Unlike the linear case, in the nonlinear region, there will be a finite bias $\Delta V$ and a temperature gradient $\Delta {\mathcal {T}}$ between two leads of the device. In the calculation, we take the right lead temperature is the same as the background temperature ${\mathcal {T}}_R={\mathcal {T}}$, so the left lead temperature is ${\mathcal {T}}_L={\mathcal {T}}+\Delta {\mathcal {T}}$. In addition, we label the chemical potentials of the left and right leads as $\mu _L$ and $\mu _R$, and $\mu _R > \mu _L$. In Eq. (7), we fixed $\mu _L$ to find the maximum power-generation efficiency $\eta$ by changing $\mu _R$ and then using Eq. (8) to calculate the equivalent figure of merit $ZT_M$. Figure 7 shows the efficiency $\eta$ and the equivalent figure of merit $ZT_M$ as functions of the chemical potential $\mu _L$ for different temperature gradient $\Delta {\mathcal {T}}$ with two magnetic domains of equal size *l* = 10 and 4.

In order to illustrate the reliability and reasonableness of the present calculations, we first compared the equivalent figure of merit $ZT_M$ calculated in the nonlinear region with the *ZT* calculated in the linear response. In Fig. 7c, the thick black lines represent *ZT* as functions of the Fermi energy $E_F$ in the linear response and the red lines represent the equivalent figure of merit $ZT_M$ of $\Delta {\mathcal {T}}$ = 0.0005 for *l* = 10 in the nonlinear region. It can be seen that the *ZT* in the linear region is fully consistent with the $ZT_M$ of $\Delta {\mathcal {T}}=0.0005$ in the nonlinear region. For *l* = 4 (see Fig. 7d), we can also draw similar conclusions. This suggests that the nonlinear $ZT_M$ regresses to the linear *ZT* when the nonlinear temperature difference $\Delta {\mathcal {T}}\rightarrow 0$. This indicates that the current calculation is completely reasonable and reliable.

Next, the thermoelectric properties in the nonlinear regime can be further explored. For *l* = 10, no resonance is present (see Fig. 2), but both $S_c$ and *ZT* are very large in the linear case [see Figs. 3a, 4a]. Thus, we take the *l* = 10, the relative tilt $\phi =\pi$, and the background temperature ${\mathcal {T}}=0.02$ in Fig. 7a,c. It is seen from the figure that as the increase of the temperature difference $\Delta {\mathcal {T}}$ from 0.0005 to 0.04, the equivalent figure of merit $ZT_M$ decreases monotonically with the $\Delta {\mathcal {T}}$, while the power-generation efficiency $\eta$ increases monotonically with $\Delta {\mathcal {T}}$. When the temperature difference $\Delta {\mathcal {T}}=0.02$, the efficiency exceeds $\eta > 40\%$, and the equivalent figure of merit $ZT_M$ also exceeds 200. Such a high equivalent figure of merit $ZT_M$ and such a large power-generation efficiency $\eta$ indicate that this devices have the necessary conditions to achieve high-performance thermoelectric power.

The equivalent figure of merit $ZT_M$ for the nonlinear region *l*= 4 is much smaller than *l*= 10, which is also similar to the case in the linear region. In Fig. 7b,d, we take the *l* = 4, the relative tilt $\phi =\pi$, and the background temperature ${\mathcal {T}}=0.05$. In the linear response, for $l=4$ , there is a resonance in the magnetic domains [see Fig. 2]. However, for the nonlinear thermoelectric transport, the equivalent figure of merit $ZT_M$ is not monotonically dependent on $\Delta {\mathcal {T}}$, and $ZT_M$ increases firstly and then decreases slightly as the temperature difference $\Delta {\mathcal {T}}$ increases from 0.0005 to 0.08. When the temperature difference $\Delta {\mathcal {T}}$=0.01, $ZT_M$ is maximum, $ZT_M\approx 95$. The power-generation efficiency $\eta$ increases with the increase of $\Delta {\mathcal {T}}$. Although the Carnot power-generation efficiency exceeds $30\%$, the equivalent figure of merit $ZT_M$ is extremely reduced when the temperature difference $\Delta {\mathcal {T}}=0.04$ and 0.08. Overall, making the efficiency $\eta > 20\%$ and the equivalent figure of merit $ZT_M > 10$, we believe that the temperature has difference $\Delta {\mathcal {T}} =0.03$ or so.

## Conclusion

In summary, the thermoelectric transport properties of helical edge states of the two-terminal nanoribbon of quantum spin Hall insulators coupling double nanomagnets have been studied, and the Seebeck coefficients $S_c$ and the thermoelectrical figure of merit *ZT*, the equivalent figure of merit $ZT_M$ and the power-generation efficiency $\eta$ for linear and nonlinear thermoelectric transport have been calculated. Because the previous paper^39^ has confirmed that it is necessary to have the device for achieving high performance thermoelectric power. We further explored the effect of the length of the nanomagnet and the relative tilt angle of component of the magnetization perpendicular on the thermoelectric transport properties, obtained the temperature difference of achieving optimal equivalent figure of merit $ZT_M$ and power-generation efficiency $\eta$. This setup (quantum spin Hall insulators coupling two nanomagnets) has a potential application as a thermoelectric device.

## Methods

### Hamiltonian and transmission function

The device coupling nanomagnet to the quantum spin Hall edge insulator is shown in Fig. 1, whose Hamiltonian can be represented as^39^,

1 $$\begin{aligned} H=\int dx\; c^{\dag }(x)[(-i \hbar v_{F} \partial _{x})\hat{\varvec{\sigma }}_{z}+J {{\textbf {m}}}(x)\cdot \hat{\varvec{\sigma }}]c(x), \end{aligned}$$

where $c(x)=(c_{R, \uparrow }(x), c_{L, \downarrow }(x))^{T}$, and $\hat{\varvec{\sigma }}=(\hat{\varvec{\sigma }}_{x}, \hat{\varvec{\sigma }}_{y}, \hat{\varvec{\sigma }}_{z})$ is the Pauli matrices. Here, the electrons with velocity $v_{F}$ and $\uparrow (\downarrow )$ spin orientation move to the right (left). *J* is the magnetic exchange interaction between the magnetic moment of the nanomagnet and the electron spin. The nanomagnet is described by the spatial distribution of the magnetic moments within the segments of lengths $L_{j}=x_{j}-x_{j-1}$^39^, so

2 $$\begin{aligned} {{\textbf {m}}}(x)=\sum _{j=1}^{N}\zeta (x_{j}-x)\zeta (x-x_{j-1}){{\textbf {m}}}_{j}, \end{aligned}$$

where ${{\textbf {m}}}_{j}=(m_{j \perp }\cos \phi _{j}, m_{j \perp }\sin \phi _{j}, m_{j \parallel })$ is a magnetic moment per unit length associated with the direction of spin-orb interaction in topological insulators, $m_{j \parallel }$ is a parallel component and $m_{j \perp }$ is a perpendicular component.

For the calculation of the transmission function, refer to Refs.^39,43^, applying the evolution operator in space throughout the scattering region, ${\hat{u}}(x_{N}, x_{0})=\Pi _{j=1}^{N}{\hat{u}}(x_{j}, x_{j-1})$. Therefore, the transmission function^39^ can be expressed as $T(E)=\mid {\textbf {Det}}[{\hat{u}}(x_{N}, x_{0})]/u(x_{N}, x_{0})_{1, 1}\mid ^{2}$, here

3 $$\begin{aligned} {\hat{u}}(x_{j}, x_{j-1})= & {} e^{\left( i\frac{E_{j \parallel }}{\hbar v_{F}}L_{j}\right) }e^{\left( -i \xi _{j} \cdot \hat{\varvec{\sigma }}\right) } \nonumber \\= & {} e^{\left( i\frac{E_{j \parallel }}{\hbar v_{F}}L_{j}\right) }\left[ \hat{\varvec{\sigma }}_{0}\cos \lambda _{j}-i{{\textbf {n}}}_{j}\cdot \hat{\varvec{\sigma }}\sin \lambda _{j}\right] , \end{aligned}$$

where $\xi _{j}=(iE_{j \perp }\sin \phi _{j}, -iE_{j \perp }\cos \phi _{j}, E)L_{j}/(\hbar v_{F})$ , with $E_{\parallel ,\perp }=Jm_{\parallel ,\perp }$ and ${{\textbf {n}}}_{j}=\frac{\xi _{j}}{\lambda _{j}}$.

In this paper, for two nanommagnets, the length is the same, while the orientation of the magnetic moment is different. So $N=2$ in Eq. (2), $L_{1}=L_{2}=L$, $\phi _{1}=0$, $\phi _{2}=\phi$ and $E_{\perp ,1}=E_{\perp ,2}=E_{\perp }$ in Eq. (3). The transmission function can be written as^39^

4 $$\begin{aligned} T(E)=\left\{ \left[ \cos ^{2}\lambda +\frac{\sin ^{2}\lambda }{r^{2}} \left( \cos \phi -\frac{E^{2}}{E_{\perp }^{2}}\right) \right] ^{2}+\left[ -\frac{E}{E_{\perp }}\frac{\sin 2\lambda }{r}+\sin \phi \frac{\sin ^{2}\lambda }{r^{2}}\right] ^{2}\right\} ^{-1}, \end{aligned}$$

here, $\lambda =rl$ and $r=\sqrt{(\frac{E}{E_{\perp }})^2-1}$, where $l=\frac{L}{L_{0}}$ and $L_{0}=\frac{\hbar v_{F}}{E_{\perp }}$.

### Thermoelectric transport

In the linear response region, the electric and heat currents are expanded linearly in a small temperature difference $\delta {\mathcal {T}}={\mathcal {T}}_L-{\mathcal {T}}_R$ and a small external bias voltage $\delta V=V_L-V_R$^14,16,17^,

5 $$\begin{aligned} \left( \begin{array}{cc} I_{L} \\ I_{L}^{Q} \\ \end{array} \right)= & {} \left( \begin{array}{cc} e^2 {\mathcal {D}}_0 &{} -e {\mathcal {D}}_1 \\ -e {\mathcal {D}}_1 &{} {\mathcal {D}}_2 \\ \end{array} \right) \left( \begin{array}{cc} \delta V \\ \delta {\mathcal {T}} /{\mathcal {T}} \\ \end{array} \right) . \end{aligned}$$

In Eq. (5), the elements of the Onsager matrix is given by ${\mathcal {D}}_i = \frac{1}{h}\int dE\; (E-E_F)^{i}(-\frac{\partial f}{\partial E})T(E)$, where $i=0,1,2$ and $f(E)=\left[ e^{(E-E_F)/k_B {\mathcal {T}}}+1\right] ^{-1}$. The transmission function *T*(*E*) can be obtained from Eq. (4).

Using Eq. (5), the linear electric conductance *G*, the Seebeck coefficients $S_c$, and the electric thermal conductance ${\kappa _{el}}$ can be expressed as^14,17^ $G = \left. \lim _{\delta V \rightarrow 0}\frac{I_L}{\delta V}\right| _{\delta {\mathcal {T}} =0}= e^{2}{\mathcal {D}}_{0}$, $S_c = -\left. \lim _{\delta {\mathcal {T}} \rightarrow 0}\frac{\delta V}{\delta {\mathcal {T}}}\right| _{I_L=0}= -\frac{1}{e{\mathcal {T}}}\frac{{\mathcal {D}}_{1}}{{\mathcal {D}}_{0}}$ and $\kappa _{el} = -\left. \lim _{\delta {\mathcal {T}} \rightarrow 0}\frac{I_{L}^{Q}}{\delta {\mathcal {T}}}\right| _{I_L=0}= \frac{1}{{\mathcal {T}}}\frac{{\mathcal {D}}_{0}{\mathcal {D}}_{2}-{\mathcal {D}}_{1}^{2}}{{\mathcal {D}}_{0}}$. We can also write the thermoelectric figure of merit $ZT=G{{S_c}^2}{\mathcal {T}}/\kappa _{el}=\frac{{\mathcal {D}}_{1}^{2}}{{\mathcal {D}}_{0}{\mathcal {D}}_{2}-{\mathcal {D}}_{1}^{2}}$, straightforwardly. Here, the lattice thermal conductance $\kappa _{ph}$ is ignored. This is because the lattice thermal conductivity is caused by the lattice vibration. At the low temperature, the lattice thermal conductance is overshadowed by the electronic thermal conductance.

In the nonlinear regime, the temperature difference $\delta {\mathcal {T}}$ and external bias voltage $\delta V$ are finite. Here set the temperature difference $\Delta {\mathcal {T}}>0$, the electric and heat currents can be written as^44–47^

6 $$\begin{aligned}&I_L= \frac{e}{h}\int dE\; T(E)[f_L(\mu _L,{\mathcal {T}}_L;E)-f_R(\mu _R,{\mathcal {T}}_R;E)], \nonumber \\&I_{\alpha }^Q = \frac{1}{h}\int dE\; (E-\mu _\alpha ) T(E)[f_L(\mu _L,{\mathcal {T}}_L;E)-f_R(\mu _R,{\mathcal {T}}_R;E)], \end{aligned}$$

where $f_{\alpha }(\mu _{\alpha },{\mathcal {T}}_{\alpha };E)=\left[ e^{(E-\mu _{\alpha })/k_B {\mathcal {T}}_{\alpha }}+1\right] ^{-1}$ is the Fermi distribution function, $\mu _{\alpha }$ is the chemical potential of ${\alpha }$-lead, with ${\alpha }=L,R$.

In this paper, the device can be seen as a power generator, so its power output *P* and efficiency of the power generator $\eta$ can be expressed as^44–47^

7 $$\begin{aligned}&P=-I_C \Delta V=\frac{1}{h}(\mu _R-\mu _L)\int dE\; T(E)[f_L(\mu _L,{\mathcal {T}}_L;E)-f_R(\mu _R,{\mathcal {T}}_R;E)], \nonumber \\&\eta =\frac{P}{I^Q_L}=\frac{(\mu _R-\mu _L)\int dE\; T(E)[f_L(\mu _L,{\mathcal {T}}_L;E)-f_R(\mu _R,{\mathcal {T}}_R;E)}{\int dE\; (E-\mu _L) T(E)[f_L(\mu _L,{\mathcal {T}}_L;E)-f_R(\mu _R,{\mathcal {T}}_R;E)]}. \end{aligned}$$

In this power generator, we set $\mu _R>\mu _L$ satisfying $P>0$. In the calculation, the temperatures ${\mathcal {T}}_{L/R}$ and chemical potential $\mu _L$ is fixed, the $\mu _R$ is changed, the maximum power-generation efficiency $\eta$ is given. Furthermore, considering the $\eta = \frac{{\mathcal {T}}_L-{\mathcal {T}}_R}{{\mathcal {T}}_L}\cdot \frac{\sqrt{1+ZT_M}-1}{\sqrt{1+ZT_M}+\frac{{\mathcal {T}}_R}{{\mathcal {T}}_L}}$^1,14^, the equivalent thermoelectric figure of merit $ZT_M$ is derived in terms of $\eta$,

8 $$\begin{aligned} ZT_M=\left[ \frac{({\mathcal {T}}_L-{\mathcal {T}}_R)+\eta {\mathcal {T}}_R}{({\mathcal {T}}_L-{\mathcal {T}}_R)-\eta {\mathcal {T}}_L}\right] ^2 -1. \end{aligned}$$

Further, we take ${\mathcal {T}}_R={\mathcal {T}}$ and ${\mathcal {T}}_L={\mathcal {T}}+\Delta {\mathcal {T}}$, ${\mathcal {T}}$ is the background temperature. Therefore, the maximum power-generation efficiency and the equivalent figure of merit can be written as^14^

9 $$\begin{aligned}&\eta = \frac{\Delta {\mathcal {T}}}{{\mathcal {T}}+\Delta {\mathcal {T}}}\frac{\sqrt{1+ZT_M}-1}{\sqrt{1+ZT_M}+{\mathcal {T}}/({\mathcal {T}}+\Delta {\mathcal {T}})}, \nonumber \\&ZT_M =\left[ \frac{\Delta {\mathcal {T}}+\eta {\mathcal {T}}}{\Delta {\mathcal {T}}-\eta ({\mathcal {T}}+\Delta {\mathcal {T}})}\right] ^2-1. \end{aligned}$$

## Data Availability

The datasets used during the current study available from the corresponding author on request. Correspondence and requests for materials should be addressed to R.W.
